# Does ethnicity affect primary palliative care identification and coding? An observational retrospective cohort study

**DOI:** 10.3399/BJGP.2025.0602

**Published:** 2026-07-28

**Authors:** Gemma Clarke, Farag Shuweihdi, Samuel D Relton, Sophie Anne Trotter, Jamilla Akhter Hussain, Matthew Allsop, Jonathan Koffman, Michael I Bennett, Sarah Mitchell, Lucy Ziegler

**Affiliations:** 1 St Gemma's Academic Unit of Palliative Care, Leeds Institute of Health Sciences, University of Leeds, Leeds, UK; 2 School of Dentistry, Faculty of Medicine and Health, University of Leeds, Leeds, UK; 3 Leeds Institute of Health Sciences, School of Medicine, University of Leeds, Leeds, UK; 4 University of Sheffield, Sheffield, UK; 5 Bradford Teaching Hospitals NHS Foundation Trust, Bradford, UK; 6 Wolfson Palliative Care Research Centre, University of Hull, Hull, UK

**Keywords:** ethnicity, general practice, palliative care, minority groups, retrospective studies, primary health care

## Abstract

**Background:**

Primary palliative care is symptom support and care for people with life-limiting illnesses incorporated within standard primary care. Early identification of needs is recommended best practice. There is limited research concerning ethnicity and primary palliative care identification and coding.

**Aim:**

To explore associations between ethnicity and primary palliative care identification and coding.

**Design and setting:**

A retrospective cohort study of deceased patients in England utilising anonymised primary care data extracted in February 2022.

**Method:**

Multilevel logistic regression was used to examine associations between ethnicity and primary palliative care records. Cox regression was used to assess time from first record to death. Differences between first palliative care codes were examined using χ^2^ tests.

**Results:**

A total of 200 876 patients were included; 32.30% (*n* = 64 887) had a primary palliative care record. Multilevel logistic regression (*n* = 64 887) showed no statistically significant association between ethnicity and identification after adjustment for age, sex, frailty, deprivation, and diagnosis. After adjustment, a mixed-effects Cox proportional hazards model (*n* = 58 681) indicated: compared with patients from White groups, patients from Asian (hazard ratio [HR] 0.944, 95% confidence interval [CI] = 0.891 to 0.999, *P* = 0.040) and Black, African, and Caribbean groups (HR 0.925, 95% CI = 0.862 to 0.992, *P* = 0.028) survived significantly longer; and patients from ‘Other’ groups had significantly shorter survival (HR 1.136, 95% CI = 1.046 to 1.234, *P* = 0.002). Patients from ethnic minority groups were significantly (*P*<0.001) more likely to have a first record of specialist palliative care compared with White groups.

**Conclusion:**

This study found no statistically significant association between ethnicity and primary palliative care identification after adjustment. However, differences were observed in survival and first palliative care activity. To ensure and monitor equitable access, ongoing improvements in palliative care identification and audits of data quality are required.

## How this fits in

As community deaths rise and primary care populations diversify, consistent recording of palliative care need is an important step towards ensuring and monitoring equitable access to primary palliative care. Primary care practices may wish to audit their palliative care and ethnicity data, paying particular attention to the ‘Other’ ethnic group category. Patients from minority ethnic groups are often reported to be disadvantaged in relation to health care in general, and palliative care access in particular. In contrast, the present study of primary palliative care recording did not suggest any such disadvantage. In this study, 32% of decedents had a primary palliative care record before death. This is consistent with another UK study, but lower than many population-based estimates of palliative care need.

## Introduction

Palliative care is defined by the World Health Organization (WHO) as *‘an approach that improves the quality of life of patients (adults and children) and their families who are facing problems associated with life-threatening illness.’* ^1^ Specialist palliative care refers to care provided by professionals with specific training or experience in palliative care.^2^ Primary palliative care refers to a type of generalist palliative care (that is, non-specialist), delivered by primary care teams to patients living at home or in community settings, such as care homes.^3^ It is a core component of general practice.^4–8^ GPs play a key role by supporting families, coordinating care, providing symptom control, and making specialist referrals.^8–10^ Understanding and improving primary palliative care is essential, as deaths in the community continue to rise in the UK and internationally,^11,12^ and home is the preferred place of death for most patients.^13^

Early access to specialist palliative care has been shown to improve quality of life and survival, and to reduce unnecessary acute care;^14–18^ however, evidence from primary palliative care is limited. Early identification and recording of palliative care needs are recommended practice within primary care,^19–21^ helping GPs to provide coordinated care aligned with individuals’ needs and preferences.^22^ Information about a patient’s palliative care needs may be clinically coded, a process by which diagnoses and procedures are transformed into standardised alphanumeric codes.^23^ Information about patients’ palliative care needs may also be recorded in ‘free text’ in their medical record. In England, the recording of patients in palliative care was previously a quality indicator in the Quality and Outcomes Framework (QOF), part of the NHS GP contract, until April 2025, when it was retired.^24,25^ However, care of the terminally unwell remains an essential service within the GP contract.^25^

Many countries have developed guidelines and standards to improve primary palliative care,^20,21,26–28^ including the UK-based Gold Standards Framework (GSF),^29^ and the Daffodil Standards developed by the Royal College of General Practitioners and Marie Curie.^20^ Both the GSF and Daffodil Standards recommend proactive early identification of primary care patients who are *‘likely to die within the next 12 months’*, and may require extra supportive care.^19,20^ The GSF offers guidance to help identification within primary care, which includes general indicators of decline, disease-specific clinical indicators, and the surprise question, *‘would you be surprised if the patient were to die in next year, months, weeks, days?’* ^19^ Despite increasing guidance and identification tools for use within primary care,^30^ identification of need remains challenging. This is, in part, because of the ambiguous nature of the term ‘palliative care need’. Population-based estimates vary substantially by definition, method, and dataset.^31–35^ Identifying patients with palliative care needs can be particularly challenging in some minority ethnic communities, where end-of-life discussions may be sensitive, and where access and engagement with services can be shaped by trust, language, and cultural norms.^36–38^ This matters increasingly as England becomes more ethnically diverse,^39^ and the prevalence of life-limiting illnesses among people from minority ethnic groups rises.^40^

There is limited research concerning ethnicity and primary palliative care identification and coding. Previous research suggests that people from minority ethnic communities are less likely to access and utilise specialist palliative care services.^41–44^ UK research on specialist palliative care referral times has been mixed; Chidiac *et al* reported patients from minority ethnic groups were identified and referred later than those from White groups,^45^ while Bajwah *et al* found no significant differences.^46^ Within the context of primary palliative care, a US-based study found ethnic inequalities in access;^47^ and a UK-based study reported ethnic disparities in opioid prescribing.^48^ Accurate identification and coding of primary palliative care status by ethnic group is essential for monitoring inequities; and to inform service planning and commissioning to ensure equitable access and culturally appropriate services.

The main aim of this study was to explore the association between patient ethnicity and primary palliative care identification and coding. The secondary aims were to explore the associations between patient ethnicity and the timing and type of first recorded primary palliative care activity codes.

## Method

### Study design

This observational retrospective cohort study of deceased primary care patients utilised routinely collected anonymised primary care and linked hospital data from England.^49^ This study is reported using STROBE (see Supplementary Appendix S1 for checklist).^50^

### Data sources and setting

Data were sourced from the Clinical Practice Research Datalink (CPRD), the largest available collection of anonymised longitudinal primary care records in England.^51^ It is broadly representative of the UK population in terms of age and sex.^51,52^ Data were extracted from CPRD Aurum in February 2022, when the data release covered 19.91% of current UK patients.^53^ Data were linked to: Hospital Episode Statistics (HES), Office for National Statistics (ONS) death registration, and Index of Multiple Deprivation (IMD).^54^ An examination of CPRD and HES-linked data found 81.7% ethnicity recording completeness.^55^

### Study cohort

The cohort comprised male or female adult (aged ≥18 years at time of death) deceased primary care patients, who died of any cause between 1 March 2018 to 29 February 2020. A 2-year sample was selected for the cohort to reduce the impact of any anomalous months or years. A most recent date of February 2020 was selected to avoid the disruption of the early COVID-19 pandemic. The date was selected at a time when the ongoing impacts of the pandemic were unknown. Patients with at least 1 day of follow-up and registration in the 24 months before death were included. Ethnicity data were extracted from CPRD and HES (where multiple records were available, the GP-coded CPRD data were preferred as most patients have more GP contact); patients with no recorded ethnicity were excluded. Ethnicity classification was based on UK census categories,^56^ in two ways:

- five groups — White; Mixed and multiple; Asian; Black, African, and Caribbean; ‘Other’; and
- two groups — White British and all other groups.
  Variable data preparation is summarised in Supplementary Table S1, and all ethnic group codes are listed in Supplementary Table S5. The baseline was ONS date of death for each patient. The study period was defined as 10 years back from baseline.

### Study variables

The main outcome of interest (‘palliative care record’) was a code of any palliative care offer or activity in the primary care record before death using CPRD data. All palliative care codes were requested from the CPRD (see Supplementary Table S6). The main outcome was subdivided into: QOF registration; other identification or code, including GSF registration, and other palliative care codes, for example, ‘terminal illness’; records of referrals to, or under the care of, specialist palliative care; advance planning, including formal advance care planning (ACP); and other records of relevant conversations (data preparation, Supplementary Table S1). The secondary outcome measure (‘time to death’) was length of survival in days between first code of palliative care activity and date of death.

Ethnicity, as described above, was the main explanatory variable. Other explanatory variables included sex, age at death, diagnosis of cancer, the electronic Frailty Index (eFI),^57^ and socioeconomic deprivation using IMD at the patient level.^58^ IMD is a composite measure of deprivation derived from geographic domains (data preparation, Supplementary Table S1).^58^

### Sample size

The sample size calculation was informed by previously reported late palliative care referral rates to ensure adequate power to explore ethnic differences in timing of referral.^59^ However, the study is retrospective and population based, and the final sample comprised all eligible deaths within the study period. Timing of referral (early/late) was analysed as an outcome within the available cohort rather than being used to restrict recruitment. The study had access to 200 876 patients in the CPRD Aurum database, which exceeds the minimum sample size of 367 patients required to estimate the late referral rate with a 5% margin of error, 80% power, and 95% confidence (based on an expected proportion of 39.5% from prior literature).^59^ This large sample size ensured robust logistic regression modelling, meeting the ‘10 events per variable’ rule for multivariable analyses.^54^

### Missing data

Across variables, 0.20% of data were missing, with 0.11% (*n* = 225) missing ethnicity data. Given the low proportion of missing data, a missing-completely-at-random assumption was made and complete case analysis undertaken.^60^ Excluding those without ethnicity may have affected the results; this is considered in the Discussion section.

### Statistical analyses

Statistical analyses were undertaken using R (version 3.4.1). Cohort sociodemographic and clinical characteristics at time of death are described using counts and percentages for categorical variables, and mean and standard deviation for continuous data. Median and interquartile range (IQR: Q1–Q3) were used for skewed continuous data. A sub-analysis of the first or only record of primary palliative care (that is, earliest code in the patient record) was undertaken using χ^2^ tests to examine associations between ethnicity and first activity codes.

#### Main outcome: palliative care record

A multilevel logistic regression model was used to examine the association between the explanatory variables and having a recorded palliative care event (yes/no), with GP practice included as a random intercept to account for clustering. The model accounted for the hierarchical structure of the data, with patients nested within GP practices. Results are reported as odds ratios with 95% confidence intervals (CIs).

#### Secondary outcome: time to death

Survival analysis was employed to investigate time to death in days by ethnicity. The start point was the date of the first palliative care record (main outcome). Any records without available data on the date of first palliative care coding were excluded from this analysis. A mixed-effects Cox regression model was used to analyse the time-to-event data and account for the nested structure (individuals nested within GPs). This model estimated hazard ratios (HRs) with 95% CIs and identified the impact of independent variables on survival. The assumption of proportional hazards was evaluated.

## Results

In total, 200 876 patients met the eligibility criteria (Figure 1). Demographic and clinical characteristics of the cohort at time of death are described in Table 1. The median patient age at death was 82.4 years (IQR 72.76–89.47), 91.98% of the cohort were White British (average of those with and without a palliative care record, which is expected given the age of the population;^56^ data not shown), 50.82% were female (Table 1), and 38.82% had a cancer diagnosis at any point.

**Figure 1.**
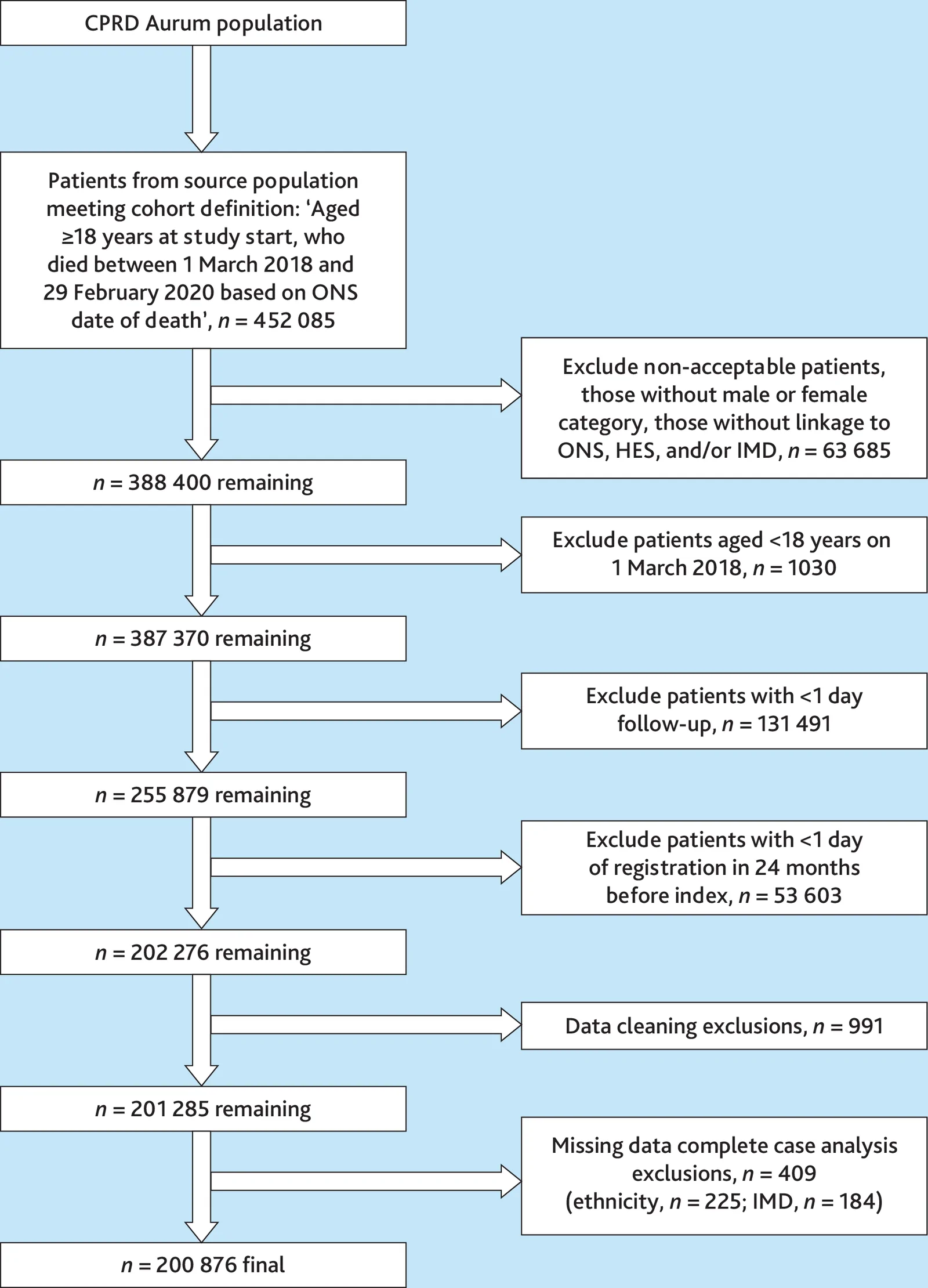
Flow diagram of study cohort inclusion. HES = Hospital Episode Statistics. IMD = Index of Multiple Deprivation. ONS = Office for National Statistics.

**Table 1.** Demographic and clinical characteristics at time of death stratified by palliative care record (*N* = 200 876)

Palliative care record, *n* (%)^a^	Yes, 64 887 (32.30)	No, 135 989 (67.70)
**Ethnicity**		
**Two-group categorisation**		
White British	60 233 (92.83)	124 530 (91.57)
All other groups	4654 (7.17)	11 459 (8.43)
**Five-group categorisation**		
White (including White British and White Other)	61 267 (94.42)	126 815 (93.25)
Asian (including South and East Asian)	1553 (2.39)	3892 (2.86)
Black, African, and Caribbean	1030 (1.59)	2650 (1.95)
Mixed and multiple	303 (0.47)	766 (0.56)
Other groups	734 (1.13)	1866 (1.37)
**Sex**		
Female	34 588 (53.30)	67 507 (49.64)
Male	30 299 (46.70)	68 482 (50.36)
**IMD group^b^**		
1 (least deprived)	21 275 (32.79)	42 297 (31.10)
2	13 262 (20.44)	26 605 (19.56)
3	18 330 (28.25)	40 143 (29.52)
4 (most deprived)	12 020 (18.52)	26 944 (19.81)
**Age at death, years, median (IQR Q1–Q3)**	82.37 (72.76–89.47)	82.51 (72.27–89.26)
**Cancer diagnosis in record**		
Yes	38 664 (59.59)	39 318 (28.91)
No	26 223 (40.41)	96 671 (71.09)
**Frailty score**		
≤0.13 (least frail)	11 793 (18.17)	30 986 (22.79)
0.14–0.25	19 592 (30.19)	41 788 (30.73)
0.26–0.34	18 775 (28.93)	38 093 (28.01)
>0.34 (Most frail)	14 727 (22.70)	25 122 (18.47)
**Frailty score average, mean (SD)**	0.25 (0.13)	0.23 (0.13)

^a^Unless otherwise specified.^ b^IMD groupings (decimal groups): 1 (1–3, least deprived); 2 (4 and 5); 3 (6–8); and 4 (9 and10, most deprived). IMD = Index of Multiple Deprivation. IQR = interquartile range.

### Palliative care record

Nearly one-third of all patients had a primary palliative care record before death (*n* = 64 887, 32.30%) (Table 1).

#### Multilevel logistic regression

After adjusting for age, sex, frailty (eFI), IMD, and cancer diagnosis, the multilevel logistic regression analysis revealed no statistically significant (*P*>0.05) association between ethnicity and any record of primary palliative care. This was the case both for the five-group (Table 2) and two-group (see Supplementary Table S2) ethnicity categorisations. Additionally, though not the primary exposures of interest, the adjusted multilevel logistic regression model indicated statistically significant associations (*P*>0.001) between primary palliative care coding and the other variables: cancer diagnosis, deprivation measures (IMD), frailty (eFI), sex, and age.

**Table 2.** Multilevel logistic regression associations between palliative care record and predictor variables by five-group ethnic categories (*N* = 200 876)

Characteristic	OR (95% CI)	*P*-value
**Intercept**	0.175 (0.161 to 0.189)	<0.001
**Ethnicity**		
White (including White British and White Other)	Reference	
Asian	1.051 (0.980 to 1.127)	0.163
Black	1.034 (0.950 to 1.125)	0.438
Mixed and multiple	1.010 (0.873 to 1.168)	0.892
Other	0.992 (0.903 to 1.090)	0.871
**Cancer diagnosis**	4.032 (3.949 to 4.116)	<0.001
**IMD group** ^**a**^		
1 (least deprived)	Reference	
2	1.015 (0.984 to 1.047)	0.359
3	0.952 (0.924 to 0.982)	0.002
4 (most deprived)	0.929 (0.895 to 0.964)	<0.001
**Frailty**		
≤0.13 (least frail)	Reference	
0.14–0.25	1.240 (1.201 to 1.279)	<0.001
0.26–0.34	1.342 (1.297 to 1.389)	<0.001
>0.34 (most frail)	1.582 (1.523 to 1.643)	<0.0001
**Sex, male**	0.824 (0.807 to 0.841)	<0.001
**Age at death**	1.003 (1.002 to 1.004)	<0.001

^a^IMD groupings (decimal groups): 1 (1–3, least deprived); 2 (4 and 5); 3 (6–8); and 4 (9 and10, most deprived). IMD = Index of Multiple Deprivation. OR = odds ratio.

#### First palliative care code

Analysis of each patient’s first or only record of primary palliative care showed many patients had multiple types of first codes (Figure 2 and Supplementary Table S3). Of those with any record (*n* = 64 887), most patients (95.78%, *n* = 62 146) had a general code describing their palliative care registration or health status. This category included GSF and other registrations, as well as health status marker codes, such as ‘terminal illness’; less than half of patients (41.12%, *n* = 26 680) had one of the QOF ‘palliative care’ registration codes. Other patients had codes indicating actions taken; 20.22% (*n* = 13 121) of patients had a record of referral to, or under the care of, specialist palliative care (including hospital and hospice); and 3.40% (*n* = 2206) had a first record of advance planning, including formal ACP and other planning conversations. Specialist palliative care codes were significantly more common in patients from minority ethnic groups compared with patients from the White group: from the five-group ethnic categorisation, as seen in Supplementary Table S3, there was over a 10% point difference between patients from Asian groups (30.78%, *n* = 478/1553) and patients from White groups (19.70%, *n* = 12 070/61 267) (*P*<0.001) (Figure 2 and Supplementary Table S3).

**Figure 2.**
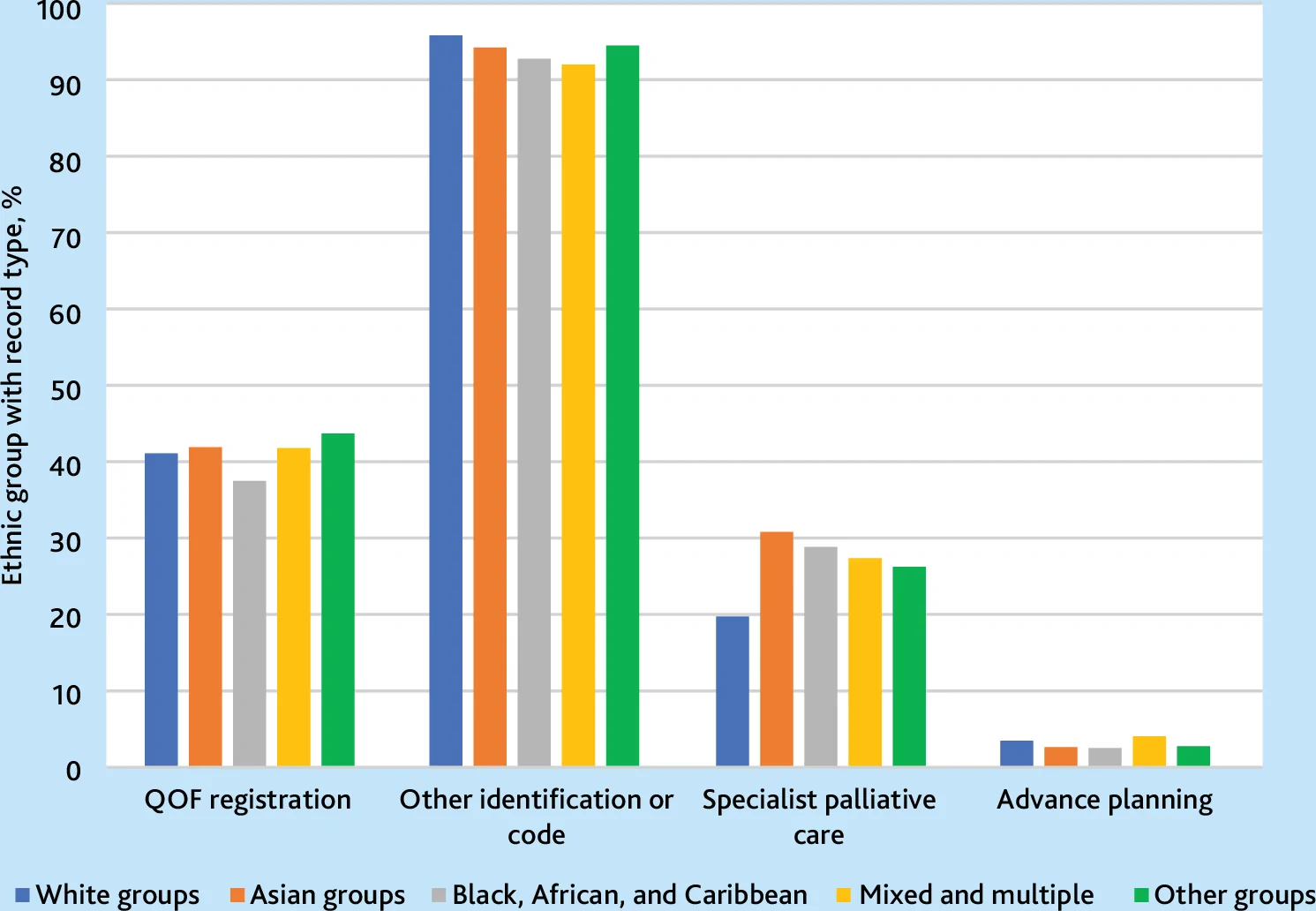
Comparison of types of patients’ first primary palliative care record stratified by ethnicity (%), using five-group ethnicity categorisation (*N* = 64 887). QOF = Quality and Outcomes Framework.

### Time to death

The time from first primary palliative care record to death was examined. Those without available data on the date of the first palliative care recording were excluded (*n* = 6206). Of those remaining with a primary palliative care record and date of first recording (*n* = 58 681): 18.31% (*n* = 10 742) of patients survived up to 30 days between first record and death; 14.28% (*n* = 8381) survived 31–90 days; 34.89% (*n* = 20 473) survived 91–365 days; and 32.52% (*n* = 19 085) survived over a year (data not shown).

#### Survival analysis

Survival analysis was undertaken to investigate variations in survival between first primary palliative care record and death. A Kaplan–Meier plot, examining survival from first record to death (*n* = 58 681), showed that patients in ‘Other’ ethnicity groups had the lowest survival probability over time (Figure 3). A mixed-effects Cox proportional hazards model (Table 3) showed that, when age, sex, frailty (eFI), deprivation (IMD), and cancer diagnosis were adjusted for, patients from Asian groups (HR 0.944, 95% CI = 0.891 to 0.999, *P* = 0.040) and Black, African, or Caribbean groups (HR 0.925, 95% CI = 0.862 to 0.992, *P* = 0.028) had statistically significantly lower hazards of death. This indicated that patients from Asian and Black, African, and Caribbean ethnic groups survived for a longer period of time after first primary palliative care recording, compared with patients from White groups. Conversely, individuals from ‘Other’ ethnic groups had a statistically significantly higher HR (1.136, 95% CI = 1.046 to 1.234, *P* = 0.002). This showed that patients from the ‘Other’ ethnicity group died significantly sooner after their first record of primary palliative care, compared with patients from White groups.

A mixed-effects Cox proportional hazards model using a two-group ethnic categorisation was also undertaken, which showed no statistically significant difference between patients from the White British ethnic group and those from all other ethnic groups (*P*>0.05) (Supplementary Table S4).

**Figure 3.**
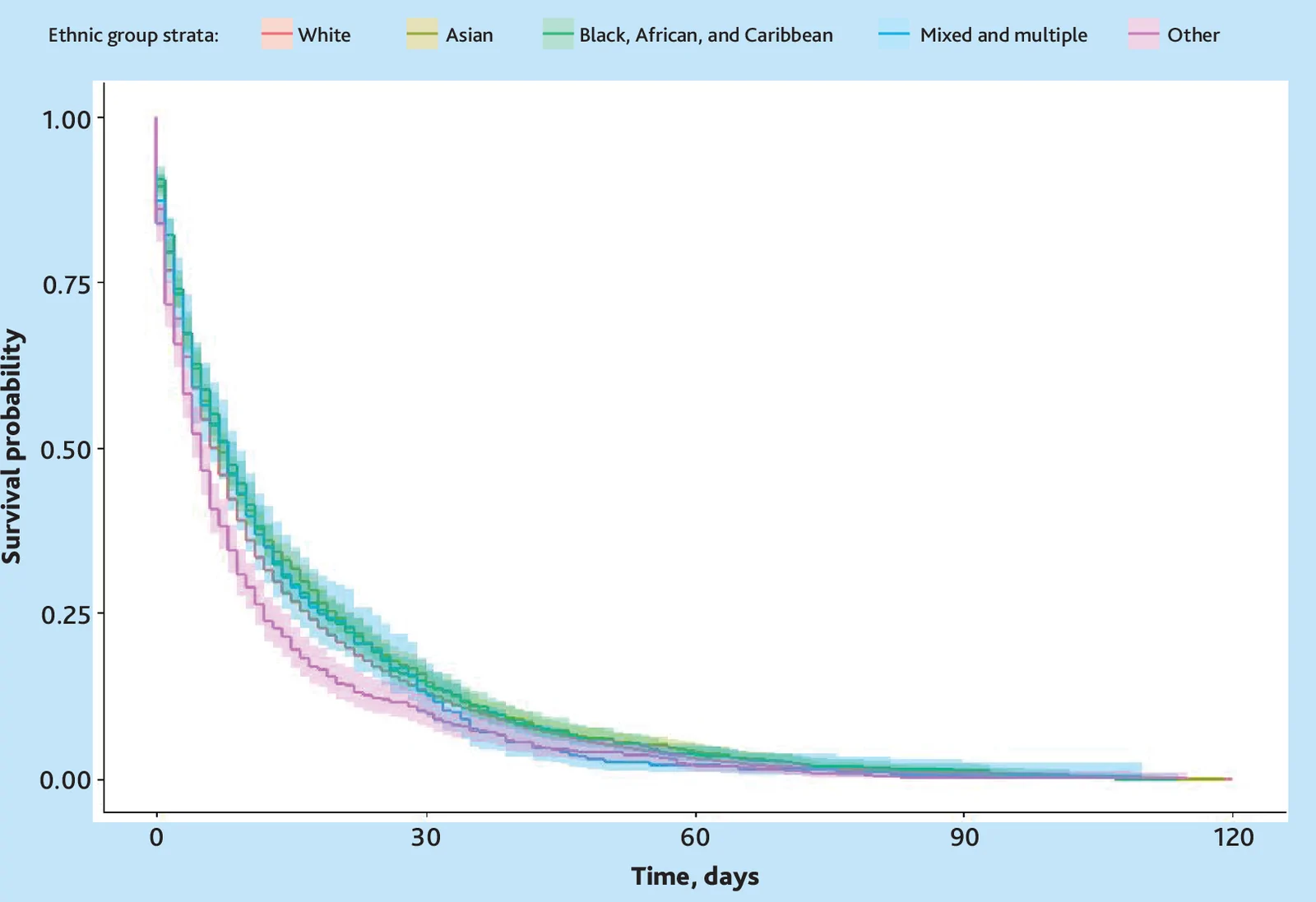
Kaplan–Meier plot for time to death from first record of primary palliative care activity, by five-group ethnic groups (*N* = 58 681).

**Table 3.** Survival analysis using Cox regression on time to death in days from first record of primary palliative care activity by five-group ethnic categorisation (*N* = 58 681)

Characteristic	HR (95% CI)	*P*-value
**Ethnicity**		
White (including White British and White Other)	Reference	
Asian (including South and East Asian	0.944 (0.891 to 0.999)	0.040
Black, African, and Caribbean	0.925 (0.862 to 0.992)	0.028
Mixed and multiple	0.979 (0.871 to 1.100)	0.072
Other	1.136 (1.046 to 1.234)	0.002
**Cancer diagnosis**		
No	Reference	
Yes	1.036 (1.012 to 1.060)	0.003
**IMD group^a^**		
1 (least deprived)		
2	0.957 (0.922 to 0.988)	0.028
3	0.955 (0.920 to 0.990)	0.007
4 (most deprived)	0.951 (0.915 to 0.988)	0.009
**Frailty**		
≤0.13 (least frail)	Reference	
0.14–0.25	0.867 (0.844 to 0.891)	<0.001
0.26–0.34	0.815 (0.791 to 0.840)	<0.001
>0.34 (most frail)	0.740 (0.713 to 0.768)	<0.001
**Sex**		
Female	Reference	
Male	1.108 (1.088 to 1.129)	<0.001
**Age at death**	1.002 (1.001 to 1.003)	<0.001

^a^IMD groupings (decimal groups): 1 (1–3, least deprived); 2 (4 and 5); 3 (6–8); and 4 (9 and10, most deprived). HR = hazard ratio. IMD = Index of Multiple Deprivation.

## Discussion

### Summary

Regression modelling indicated no statistically significant association between ethnicity and primary palliative care identification and coding, after adjustment for age, sex, frailty, deprivation, and cancer diagnosis. Overall, nearly one-third (32%) of patients had a primary palliative care record before death (*n* = 64 887). After identification, around one-third of patients survived for up to 90 days, around one-third survived between 91 days and 1 year, and around one-third survived over 1 year. Patients from minority ethnic groups were significantly more likely to have a first record of specialist palliative care activity, compared with patients from White groups. After adjustment, a mixed-effects Cox proportional hazards model (*n* = 58 681) indicated that, compared with patients from White groups, patients from Asian, Black, African, or Caribbean groups survived significantly longer from first record to death, and patients from ‘Other’ groups had significantly shorter survival times.

This study found that patients from minority ethnic groups do not appear disadvantaged in accessing primary palliative care support before death compared with patients from White groups. However, differences were observed in survival for some minority ethnic patients, and patients from minority ethnic groups were more likely to have a first record of specialist palliative care. Around one-third of patients had a primary palliative care record, which is substantially lower than population-based estimates of palliative care need, potentially reflecting inadequacies in identification and/or coding.

### Strengths and limitations

This is the first national study to examine the relationship between ethnicity and primary palliative care identification and coding in England using routine data, which is underutilised in palliative care.^49^ There are a number of limitations. Those without a recorded ethnicity were excluded, which may have affected the results. Using only two- and five-group ethnicity categorisations may have obscured intragroup variability.^61^ This study relies on clinical coding. Clinical coding may not always be accurate and complete,^62^ and there may be additional challenges for clinical coding within primary care.^63^ However, QOF codes, such as ‘palliative care’ during the period of this study, are more complete than other primary care clinical codes.^64^ Ethnic group data codes may also be inaccurate or incomplete.^61^ Clinical codes do not show the full picture of a patient’s care. Information about a patient’s palliative care needs may not be coded, but instead recorded qualitatively in the ‘free text’ of a patient’s medical record. It is unknown who input data. Primary care data may have been input by specialist palliative care teams, which could have affected the results, as coding practices between specialist and primary care may differ. The multilevel logistic regression model accounted for the hierarchical structure of data, with patients nested within GP practices. However, it was not confirmed whether any patients switched GP practices. The eFI^57^ is a composite measure, combining multiple deficits from electronic health records. Adjusting for eFI could lead to model overfit, which could have affected results. Additionally, the dataset is pre-COVID-19 and primary care practice has since changed in the UK.^65,66^

### Comparison with other literature

After model adjustment, there was no statistically significant association between ethnicity and primary palliative care identification and coding. This finding is in contrast with much of the UK and international literature on ethnicity and specialist palliative care, which has shown ethnic disparities in access to services,^43,67–71^ prescribing,^48,72^ advance planning,^71,73,74^ and use of acute care at the end of life.^48,71^ The reasons why the findings from this study differ from those observed in specialist palliative care are unknown. It may reflect a real difference in equity of access to care. In the UK, primary care has a universal point of access, and therefore fewer barriers and less gatekeeping. It could also be the result of better recording practices, due to incentivised coding practices within primary care. The recording of ‘palliative care’ patients was previously an incentivised quality indicator, part of the NHS GP contract until April 2025.^24,25^ However, as discussed, clinical coding in primary care can be challenging and may not always be complete. Additionally, correct identification and coding do not necessarily translate into patient experience. Bajwah *et al*’s study into specialist palliative care services during the COVID-19 pandemic found services were equal on paper, but inequitable in practice; patients from minority ethnic groups may have experienced adverse impacts such as communication challenges and unmet faith needs.^75^ The provision of primary palliative care is a complex multi-component intervention, of which, identification and coding are only partial components. Further research is required, including realist methods to understand and evaluate the complex mechanisms of providing equitable high-quality care.

Though there was no association with ethnicity, other sociodemographic and disease factors may be important. While not the primary exposures of interest, the regression modelling indicated some significant associations between primary palliative care and cancer diagnosis, deprivation, frailty, sex, and age. Evidence suggests there may be relationships between these variables and healthcare access;^43,76–81^ future research could investigate these factors and their relationships to primary palliative care access.

Patients from minority ethnic groups were more likely to have a first primary palliative care record of specialist palliative care. One explanation might be hesitancy to access primary care, in line with UK research showing that patients from minority ethnic groups report poorer experiences of primary care.^82^ Another possible explanation could be first presentation in hospital, or late disease presentation,^83,84^ but this is at odds with the current study’s finding that patients from Asian, Black, African, or Caribbean groups survived longer than those from White groups. Rather, these findings reflect those from another UK study, which reported patients from Black, African, Caribbean, and Black British groups had the longest survival between hospice referral and death, compared with other groups.^59^

A UK hospital data study found ‘Other’ ethnicity was poorly and inconsistently recorded during short periods of urgent care, and suggested patients may be coded as ‘Other’ when their ethnicity is unknown.^85^ This may be reflected in the findings from this study, in which patients from ‘Other’ ethnic groups had the shortest survival times. This is particularly pertinent as levels of urgent care use at the end of life are high; 61% of people in England have ≥1 emergency admissions in the last year of life.^86^

This study showed 32% of patients had a primary palliative care record before death; this reflects a smaller-scale study of primary palliative care registration in Scotland, which found 29% of patients had a record.^87^ These figures are substantially lower than most population-based estimates of palliative care need.^31–35^ This potential gap could result from factors including lack of resources, inadequate identification tools, or low GP skills and confidence.^30,88,89^ It may also be the result of inconsistent or poor recording, as a UK survey reported 86% of patients had primary care contact in the months before death.^90^ It could also result from challenges in identifying need in some communities, where end-of-life discussions are sensitive, such as frail older people, those with non-malignant disease, and people from minority ethnic communities.^8,36–38^ More research understanding the complex processes of identifying palliative care need within primary care is required. Additionally, patients may receive specialist palliative care elsewhere, such as in a hospital or hospice, which should be communicated to primary care. Research shows hospice and hospital discharge letters frequently lack required information.^91,92^

### Implications for research and practice

To ensure and monitor equitable access to primary palliative care, ongoing improvements in the identification of patients’ palliative care needs are required. Primary care practices may wish to audit their palliative care coding and their ethnicity data, particularly the ‘Other’ ethnic category. This is particularly pertinent because of the increasing number of community-based deaths. This could be enabled by contractual incentives and robust, validated tools to support the identification process to align with population need and address inequity.
Further research is required to understand the patient experience of identification, impact on access to palliative care, and the relationships between ethnicity, primary palliative care, and other potentially important factors such as socioeconomic status, sex, and frailty. Realist methods could be used to explore and evaluate the complex mechanisms of providing equitable high-quality care.

## Data

Data are available on application to CPRD: https://www.cprd.com
